# Therapies for people hospitalized with COVID-19 and alignment with national clinical guidelines in a large hospital, Almaty, Kazakhstan, 2020–2021

**DOI:** 10.3389/fmed.2023.1248959

**Published:** 2023-09-21

**Authors:** Saya Gazezova, Dilyara Nabirova, Ariana Detmar, Manar Smagul, Lena Kasabekova, Sanam Zikriyarova, Roberta Horth

**Affiliations:** ^1^Central Asia Field Epidemiology Training Program, Asfendiyarov Kazakh National Medical University, Almaty, Kazakhstan; ^2^Scientific and Practical Center for Sanitary and Epidemiological Expertise and Monitoring, Almaty, Kazakhstan; ^3^Division of Global Health Protection in Central Asia, United States Centers for Disease Control and Prevention, Almaty, Kazakhstan; ^4^Health Sciences Unit, Faculty of Social Sciences, Tampere University, Tampere, Finland; ^5^Division of Global Health Protection, United States Centers for Disease Control and Prevention, Atlanta, GA, United States

**Keywords:** COVID-19, coronavirus infection, clinical guidance, Kazakhstan, antibiotic use

## Abstract

**Background:**

Clinical practice guidelines were continually changing during the COVID-19 pandemic to reflect the best available evidence for a novel virus. In Kazakhstan, the national clinical guidelines for COVID-19 patient care were regularly modified and it was not known if and to what extent these guidelines were being followed in practice.

**Methods:**

We conducted a sub-analysis of data collected from an observational study among people hospitalized with COVID-19 in a large infectious disease hospital in Almaty in four cross-sections of increased COVID-19 incidence: T1 (1 June–30 August 2020); T2 (1 October–31 December 2020); T3 (1 April–31 May 2021); and T4 (1 July–26 October 2021). Modifications to the national COVID-19 treatment guidelines were identified and clinical data were abstracted from electronic medical records. We assessed frequency of antibiotic, glucocorticoid, anticoagulant, and antiviral administered in each period and determined if these aligned with national clinical guidelines. We used multivariable logistic regression to compare practices across periods.

**Results:**

Six modifications were made to national COVID-19 treatment guidelines during this study. Of 1,146 people hospitalized with COVID-19, 14% were in T1, 14% in T2, 22% in T3, and 50% in T4. Anticoagulant treatment was administered to 87% (range: 56%–95%), antibiotic treatment to 60% (range: 58%–64%), glucocorticoid to 55% (range: 43%–64%) and antiviral therapy 15% (range: 7%–22%). Majority of treatments were not aligned with national guidelines, including 98% of anticoagulant use, 95% of antibiotic use, 56% of glucocorticoid use, and 56% of antiviral use. There were no significant changes in practice following changes in guidelines for antibiotic use (64% in T1 to 58% in T2, *p* = 0.30). There was significant increase in use of anticoagulant (84% in T2 vs. 95% in T3, *p* < 0.01), glucocorticoid (43% in T2 vs. 64% in T3, *p* < 0.01), and antiviral treatment (7% in T3 vs. 15% in T4, *p* < 0.01) after guidelines updates.

**Conclusion:**

The majority of treatments administered to people hospitalized with COVID-19 in four periods of high incidence in Almaty were not aligned with updated clinical guidelines. Antibiotic misuse was markedly high throughout. Increased awareness and training on clinical practice guidelines as updates are released may help improve adoption of evidence-based practices.

## Introduction

The novel coronavirus disease 2019 (COVID-19) pandemic had a large impact on human health which strained healthcare systems around the world. As of 27 July 2023, over 768 million confirmed COVID-19 case-patients and 6.9 million deaths were reported globally (1). In response to an ever-changing evidence base and understanding of COVID-19 epidemiology, countries and health organizations across the world have had to create and frequently adapt hundreds clinical practice guidelines for prompt and effective COVID-19 care (2–8). The World Health Organization regularly updated their guidance for COVID-19 clinical practice to reflect the most recent science (9). However, national guidelines often do not change rapidly enough to reflect the latest scientific evidence (10). Also, practitioners may be slow to learn about changes in clinical guidelines and adopt these in practice. A study in the Netherlands found that healthcare providers follow clinical practice guidelines in only about 67% of time (11).

In Kazakhstan, 1.5 million COVID-19 cases and 19,072 deaths were reported as of 27 July 2023 (12). The country was adapting to changing pandemic with over 2,000 public health decisions and 83 resolutions of the Chief State Sanitary Doctor on sanitary, preventive, and anti-epidemic measures developed. Sixteen modular hospitals dedicated solely to COVID-19 were built in 12 out of 17 oblasts and 3 cities with highest COVID-19 incidence. Additionally, three infectious diseases hospitals were renovated for hospitalization of confirmed and probable COVID-19 cases, and over 63 outpatient healthcare facilities were put into operation to provide ambulatory care for COVID-19 patients (13). Over 3,054 mobile teams provided medical care at home for COVID-19 patients. COVID-19 treatment in the country is provided to patients free of charge both in hospitals and in outpatient facilities. The national clinical management guidance for diagnosis and treatment of COVID-19 was updated 15 times after first release in February 2020 until July 2023 (14). Adoption of these recommendations by clinicians has never been investigated in Kazakhstan.

Timely adoption clinical guideline updates during a pandemic response including of the most up-to-date clinical recommendations published in guidelines are essential to reduce morbidity and mortality in patients with COVID-19. Understanding if and to what extent best practices for patient care were adopted by clinicians is essential as we enter the recovery phase of the pandemic response and prepare lessons learned for future disease threats. The purpose of this study is to describe clinical management practices of clinicians treating hospitalized COVID-19 patients in Almaty, Kazakhstan and to assess these treatments with respect to the changing national clinical guidance during the pandemic.

## Methods

### Study design

**Figure 1:**
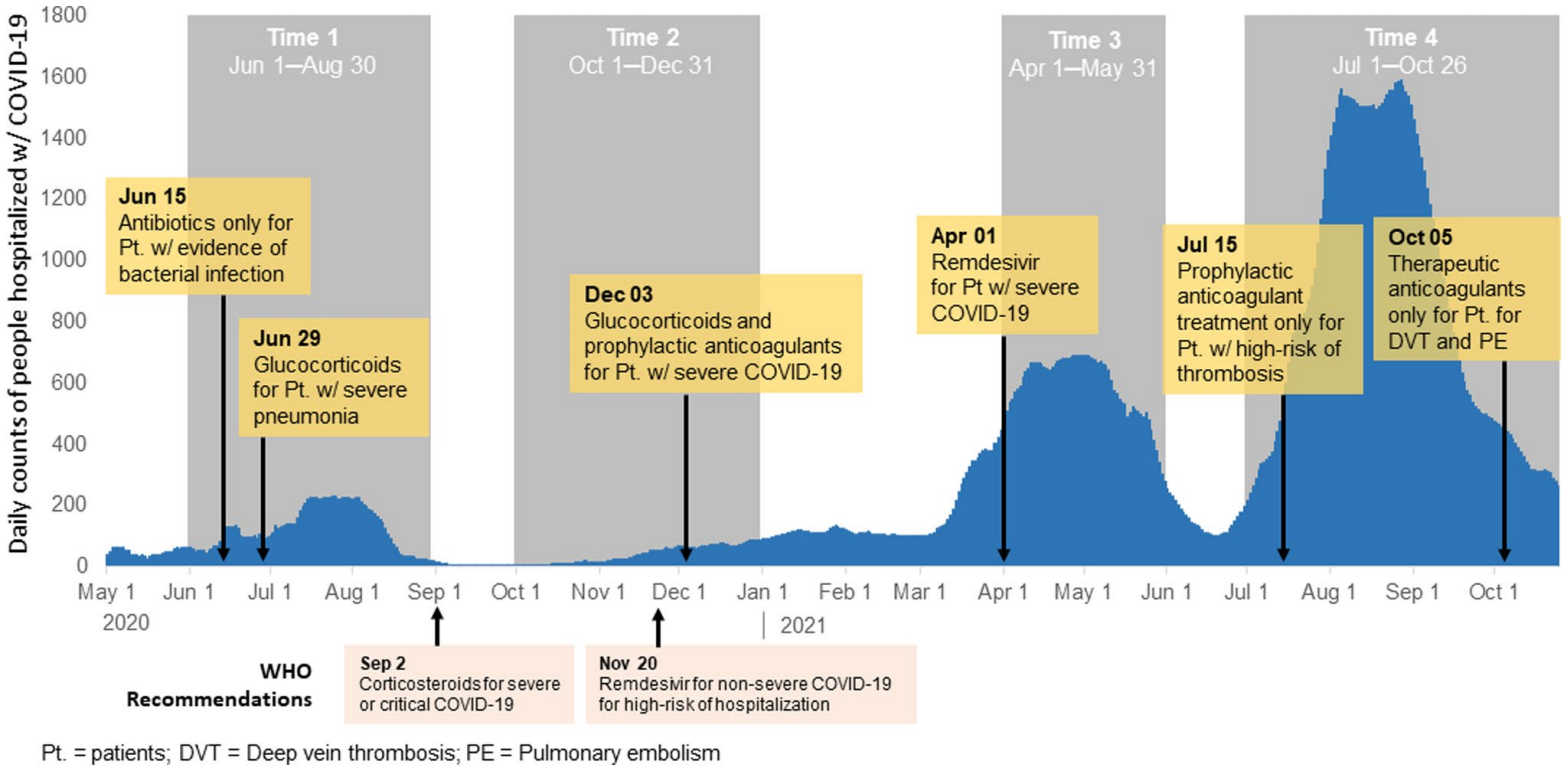
Modifications to the national clinical protocol for diagnosis and treatment of COVID-19 and histogram of COVID-19 cases in Almaty, Kazakhstan, 2020–2021.

Our study is a secondary analysis of data from an observational study of people 18 years or older hospitalized with COVID-19 in an adult infectious disease hospital in Almaty in four time periods of increased COVID-19 incidence: T1 (1 June–30 August 2020); T2 (1 October–31 December 2020); T3 (1 April–31 May 2021); and T4 (1 July–26 October 2021; Figure 1). The periods were selected during four waves where daily COVID-19 cases were high at a national level.

### Study setting

Almaty is the largest city in Kazakhstan with 1.3 million adults. COVID-19 treatment in the country is provided to patients free of charge both in hospitals and in outpatient facilities. The infectious disease hospital where we conducted the study is a specialized Almaty City facility for treating various infectious diseases, including COVID-19 in adults 18 years or older. During the COVID-19 pandemic this was the primary tertiary referral public hospital for caring for COVID-19 patients. It had 3 campuses dispersed geographically in different districts of the city to accommodate high volume of care for people with COVID-19.

### Participant selection

Study participants were selected using systematic sampling where every 14th person admitted was selected from the 17,400 people hospitalized with COVID-19 in infectious disease hospital in the four periods of the study. This population was divided into 4 waves reported in Kazakhstan during the study period. The number of adults hospitalized with COVID-19 in the each of the four waves was 2517, 2360, 3779, and 8744, respectively. We used sampling proportional to size for each wave. Assuming 17400 population size and 95% confidence level, sample size of 1146 would be sufficient to have a margin of error of 0.028 or 2.8% with an expected mortality rate was 0.5 (conservative estimate) (1).

### Key definitions

A COVID-19 case was defined using national case definition from this period.

- Confirmed COVID-19 case was defined as people with laboratory confirmation of COVID-19 by PCR, regardless of clinical signs and symptoms.
- Probable cases were defined as patients lacking confirmatory laboratory evidence for SARS-CoV-2, with typical chest imaging examination findings indicative of COVID-19 that met any of the following: (1) received treatment at a medical facility within the past 14 days where a case of COVID-19 was reported, (2) worked in health care settings, including inpatient and outpatient settings within the 14 days prior to the onset of symptoms, (3) had any acute respiratory illness (ARI) with a history of exposure to a confirmed or probable case of COVID-19 within 14 days prior to onset of symptoms, (4) had any acute respiratory infection (ARI) and pneumonia of unspecified etiology, in addition to three or more of the following symptoms: fever, cough, general weakness/fatigue, headache, myalgia, sore throat, runny nose, shortness of breath, anorexia/nausea/vomiting, diarrhea, change in mental status.

Severe COVID-19 was defined in medical records using the Ministry of Health (14, 15) as follows:

1. Signs of pneumonia (fever, cough, shortness of breath), plus
  - Pulmonary infiltrates on chest x-ray or computer tomography imaging.
  - Respiratory rate ≥ 30 breaths per minute,
  - Acute respiratory distress syndrome or SpO2 < 90% on room air requiring:
    - Admission to intensive care unit and
    - Oxygen supplementation and mechanical ventilation (16).
  - Other signs of severe COVID-19 included thromboembolism, sepsis and septic shock and/or
  - Multi-organ failure including acute kidney injury, cardiac injury and encephalopathy (17).

### Data sources

Clinical data that is stored in unstructured format as notes in patient medical charts such as dates of admission, demographic variables, comorbidities, ICD codes (for COVID-19 diagnosis), symptoms and symptom onset, laboratory testing, and therapies and medications administered were abstracted from hospital records by trained Field Epidemiology Training Program residents and epidemiologists. Data was abstracted in July-August 2022, and data validation and cross-checking was performed. Missing data was searched for in patient medical charts.

Treatment compliance with clinical guidelines was determined for each treatment type for each hospital record. Modifications to national clinical practice guidelines, referred to as guidelines hereafter, for the diagnosis and treatment of COVID-19 during the four time periods were recorded and categorized by the following treatments anticoagulants, antivirals, glucocorticoids, and antibiotics, referred to as treatment hereafter (Supplementary Table 1). For each patient who received any of these four treatments in the time periods after guidelines had been added for that treatment, we determined whether or not patients had met the criteria for receiving the specific treatment. Alignment with guidance was defined by treatment type as:

- Antibiotic treatment only for patients with evidence of bacterial infection.
- Glucocorticoids treatment only for patients with severe COVID-19.
- Antiviral treatment only for patients with severe COVID-19 or risk factors for severe COVID-19 based on age and comorbid conditions.
- Anticoagulant treatment prophylactically only for patients with severe COVID-19 or therapeutically for patients with thrombosis or pulmonary embolism in T2 and T3. In T4, therapeutic treatment only for patients that had signs of thrombosis or pulmonary embolism.

Comorbidity was defined as having any of the following conditions: obesity, hypertension, diabetes mellitus, cardiovascular disease, chronic heart failure, chronic obstructive pulmonary disease, ischemic heart disease without coronary intervention, kidney disease, hypotension, acute heart failure, acute coronary syndrome, ischemic cardiomyopathy at the time of treatment with COVID-19, encephalopathy, acute kidney injury or acute renal failure.

### Ethical considerations

Ethical approval of the study was received from the local Ethical Commission of the NAO Kazakh National Medical University named after S.D. Asfendiyarov, Kazakhstan [No. 6 (129), 05/25/2022]. This activity was reviewed by the CDC and was conducted consistently with applicable U.S. federal law and CDC policy.

### Statistical analysis

Data cleaning and analysis were performed in R v.4.2.1. Standard summary statistics were used to describe characteristics of patients (sex, age, and comorbidities) and treatments during each of the four periods. To assess trends in proportion of treatment types administered across all periods, with time as a continuous variable, we used quasi-binomial logistic regression controlling for sex, age, comorbidity, severe COVID-19, and vaccination status. We also separately used Cochran-Armitage trend test to assess changes in proportion of treatments administered that were in alignment with national guidelines across T2 to T4 periods. Missing data was treated as a missing category rather than excluded from analysis. *P*-values of <0.05 are considered significant.

## Results

There were six updates made to the national clinical management protocol for the diagnosis and treatment of COVID-19 were made during the study period (Figure 1; Supplementary Table 1). The first update occurred during T1 on 15 June 2020 and recommended use of antibiotics only for patients with secondary infection. During T1 a second update was made to recommend use of glucocorticoids for patients with severe pneumonia. The next update occurred in T2 (December 3, 2020) when glucocorticoids were recommended for all patients with severe COVID-19. This update also include recommendation for prophylactic dose anticoagulants (heparin-based) for all patients with severe COVID-19. In T3 on 1 April 2021, an update was made to recommend antiviral medications (Remdesivir) for patients with severe COVID-19 or those with risk factors for severe disease though there was limited availability of Remdesivir in Kazakhstan during this time. There were two changes that occurred in T4. On 15 July 2021, guidelines recommended prophylactic anticoagulant therapy only for hospitalized patients with COVID-19 with signs of thrombosis. Lastly, on 5 October 2021, therapeutic anticoagulant use was recommended for patients with COVID-19 with deep vein thrombosis or pulmonary embolism.

Our study included 1,146 people hospitalized during the four periods. Of these 59% (676) were female and mean age was 57 years old (range 18–96 years; Table 1). Half (51%) of patients were <60 years old, 26% were obese, 64% had a comorbidity, 34% had severe COVID-19, and 10% died. The distribution of patients across the four periods, T1 to T4 respectively, was 14% (165), 12% (141), 22% (256), and 51% (584), respectively. Across the periods, there was a significant (*p* < 0.01) increase in proportion of patients that were ≥60 years old, that had obesity, comorbidity, severe disease, and that died.

**Table 1:** Characteristics of people hospitalized with COVID-19, Kazakhstan, 2020–2021.

Characteristics	Overall	T1	T2	T3	T4	*P* ^**^
	*n* (%)	*n* (%)	*n* (%)	*n* (%)	*n* (%)	
Total *N* (%)	1,146 (100)	165 (14)	141 (12)	256 (22)	584 (51)	
**Sex**						
Male	676 (59)	89 (54)	81 (57)	152 (59)	354 (61)	0.12
Female	470 (41)	76 (46)	60 (43)	104 (41)	230 (39)	
**Age category**						
<60	585 (51)	104 (63)	89 (63)	120 (47)	272 (47)	<0.01
≥60	561 (49)	61 (37)	52 (37)	136 (53)	312 (53)	
**Obese**						
No	737 (64)	140 (85)	84 (60)	154 (60)	359 (62)	<0.01
Yes	303 (26)	19 (12)	13 (9)	73 (29)	198 (34)	
(Missing)	106 (9)	6 (4)	44 (31)	29 (11)	27 (5)	
Have comorbidities^*^						
No	411 (36)	82 (50)	71 (50)	99 (39)	159 (27)	<0.01
Yes	735 (64)	83 (50)	70 (50)	157 (61)	425 (73)	
**Have severe COVID-19**						
No	762 (67)	145 (88)	111 (79)	159 (62)	347 (59)	<0.01
Yes	384 (34)	20 (12)	30 (21)	97 (38)	237 (41)	
**Vaccinated against COVID-19**						
No	1,033 (90)	161 (98)	134 (95)	246 (96)	492 (84)	<0.01
Yes	109 (9)	0 (0)	0 (0)	7 (3)	92 (16)	
(Missing)	4 (0.3)	0 (0)	1 (0.7)	3 (1)	0 (0)	
**Died**						
No	1,033 (90)	156 (95)	135 (96)	232 (91)	510 (87)	<0.01
Yes	113 (10)	9 (5)	6 (4)	24 (9)	74 (13)	

T1 (1 June–30 August 2020); T2 (1 October–31 December 2020); T3 (1 April–31 May 2021); and T4 (1 July–26 October 2021). ^*^Including immunocompromised, hypertension, diabetes, cardiovascular disease, congestive heart failure, kidney disease, asthma, obesity. ^**^Wald-test from quasi-binomial logistic regression.

### Frequency and trends in treatments

Of the four treatment types assessed, anticoagulant treatment was the most administered medication across the four waves with 87% (995) of patients receiving them (Table 2). There was an increasing trend (*p* < 0.01) in the proportion of participants that received anticoagulant treatment across the periods. Also, there was significant change in use of anticoagulant from T2 [when they were first recommended in guidelines to the next period T3 (84% vs. 95%, respectively, *p* < 0.01)].

Antibiotics were administered to 60% (686) (range: 58%–64% across the periods). Adjusted trend analysis shows increasing proportion of participants received antibiotics over time. There was no change in antibiotic use after the guideline update in T1 recommending their use only for secondary infections (64% in T1 to 58% in T2). Cephalosporin antibiotics were the most commonly administered antimicrobials across the 4 periods (78%, 64%, 71%, and 67%, respectively) followed by fluoroquinolones (36%, 32%, 32%, and 35%, respectively, *p* < 0.01).

Glucocorticoid were administered to 55% (626) of participants (range: 43%–56%). Glucocorticoid use increased to 64% in T3 from 43% in T2 when guidelines were updated recommending their use for persons hospitalized with severe COVID-19. The use of glucocorticoid significantly dropped to 56% (*p* < 0.01) in T4 though there were no additional changes to guidance related to their use during this time. Lastly, 15% (174) of participants (range 7%–22%) were given antivirals (only during T3 did Remdesivir become available in country before T3 the primary antiviral used was lopinavir-ritonavir). Use of antivirals decreased (*p* < 0.01) from 22% in T1 to 7% in T3, but significantly increased (*p* < 0.01) to 15% in T4 following recommendations that had been made for their use during T3.

**Table 2:** Treatments administered to people hospitalized with COVID-19, Kazakhstan, 2020–2021.

Treatments administered	Overall	T1	T2		T3		T4		Crude	Adjusted
	*N* (%)	*n* (%)	*n* (%)	*P* ^*^	*n* (%)	*P* ^*^	*n* (%)	*P* ^*^	*P* ^**^	*P* ^***^
Anticoagulant treatment				**<0.01**		**<0.01**		0.11	**<0.01**	**<0.01**
Yes	995 (87)	93 (56)	118 (84)		243 (95)		541 (92)			
No	152 (13)	72 (44)	23 (16)		13 (5)		43 (7)			
Antibiotic treatment				0.30		0.834		0.07	0.28	**<0.01**
Yes	686 (60)	104 (64)	82 (58)		162 (63)		338 (58)			
No	461 (40)	61 (37)	59 (42)		94 (37)		246 (42)			
Glucocorticoid treatment				0.54		**<0.01**		**<0.01**	**<0.01**	0.66
Yes	626 (55)	76 (46)	61 (43)		165 (64)		324 (56)			
No	521 (45)	89 (54)	80 (57)		91 (36)		260 (45)			
Antiviral treatment				**<0.01**		**0.05**		**<0.01**	**<0.01**	**<0.01**
Yes	174 (15)	53 (22)	17 (12)		17 (7)		87 (15)			
No	973 (85)	112 (68)	124 (88)		239 (93)		497 (85)			

T1 (1 June–30 August 2020); T2 (1 October–31 December 2020); T3 (1 April–31 May 2021); and T4 (1 July–26 October 2021). Grayed boxes represent changes made to national clinical practice guidelines in that period; bolded represent *p*-values < 0.05. ^*^Wald-test from generalized linear model adjusting for sex, age, comorbidity, severe COVID-19 and vaccination status comparing current to previous time period. ^**^Wald-test from quasi-binomial logistic regression with no covariables to assess trend across all periods. ^***^Wald-test from quasi-binomial logistic regression adjusting for sex, age, comorbidity, severe COVID-19 and vaccination status to assess trend across all periods.

### Treatment alignment with national guidance

Of the treatments administered, a large proportion were not aligned with national guidelines. Of the 784 patients who received anticoagulant treatment in T3 and T4 (after guidance had included information on anticoagulant use), it was not in alignment with guidance for the 98% of patients that received it (Table 3). Among the 582 patients who received antibiotic treatment in T2 to T4 (after guidance for antibiotic use had been published), 95% of patients who received it had no signs or diagnosis of bacterial infection. Of the 550 patients that received glucocorticoid in T2 to T4, 53% did not meet clinical guidelines criteria. Lastly, of the 104 patients who received antiviral treatment, 56%did not meet criteria. The exception of glucocorticoid, there was no increasing trend in alignment with national guidelines over time.

**Table 3:** Alignment of treatments administered with national clinical guidelines among people hospitalized with COVID-19, Almaty, Kazakhstan, 2020–2021.

Treatments administered in alignment ^*^ with national guidelines	Overall^**^	T2	T3	T4	*P* ^***^
	*n* (%)	*n* (%)	*n* (%)	*n* (%)	
Anticoagulant (*n* = 784)					0.68
Aligned	17 (2)	NA	4 (2)	13 (2)	
Not	767 (98)	NA	239 (98)	528 (98)	
Antibiotic (*n* = 582)					0.25
Aligned	28 (5)	6 (7)	8 (5)	14 (4)	
Not	554 (95)	76 (93)	154 (95)	324 (96)	
Glucocorticoid (*n* = 550)					<0.01
Aligned	260 (47)	17 (28)	80 (49)	163 (50)	
Not	290 (53)	44 (72)	85 (52)	161 (50)	
Antiviral (*n* = 104)					
Aligned	46 (44)	NA	8 (47)	38 (44)	0.80
Not	58 (56)	NA	9 (53)	49 (56)	

T1 (1 June–30 August 2020); T2 (1 October–31 December 2020); T3 (1 April–31 May 2021); and T4 (1 July–26 October 2021). NA, treatment not specified in national guidelines. ^*^Cochran-Armitage trend test. ^*^Alignment refers to having received the treatment and meeting criteria as per guidelines and not aligned means having received the treatment but not meeting criteria as per guidelines. ^**^The denominators are patients who received treatment after guidelines were developed. ^***^Cochran-Armitage trend test.

**Figure 2:**
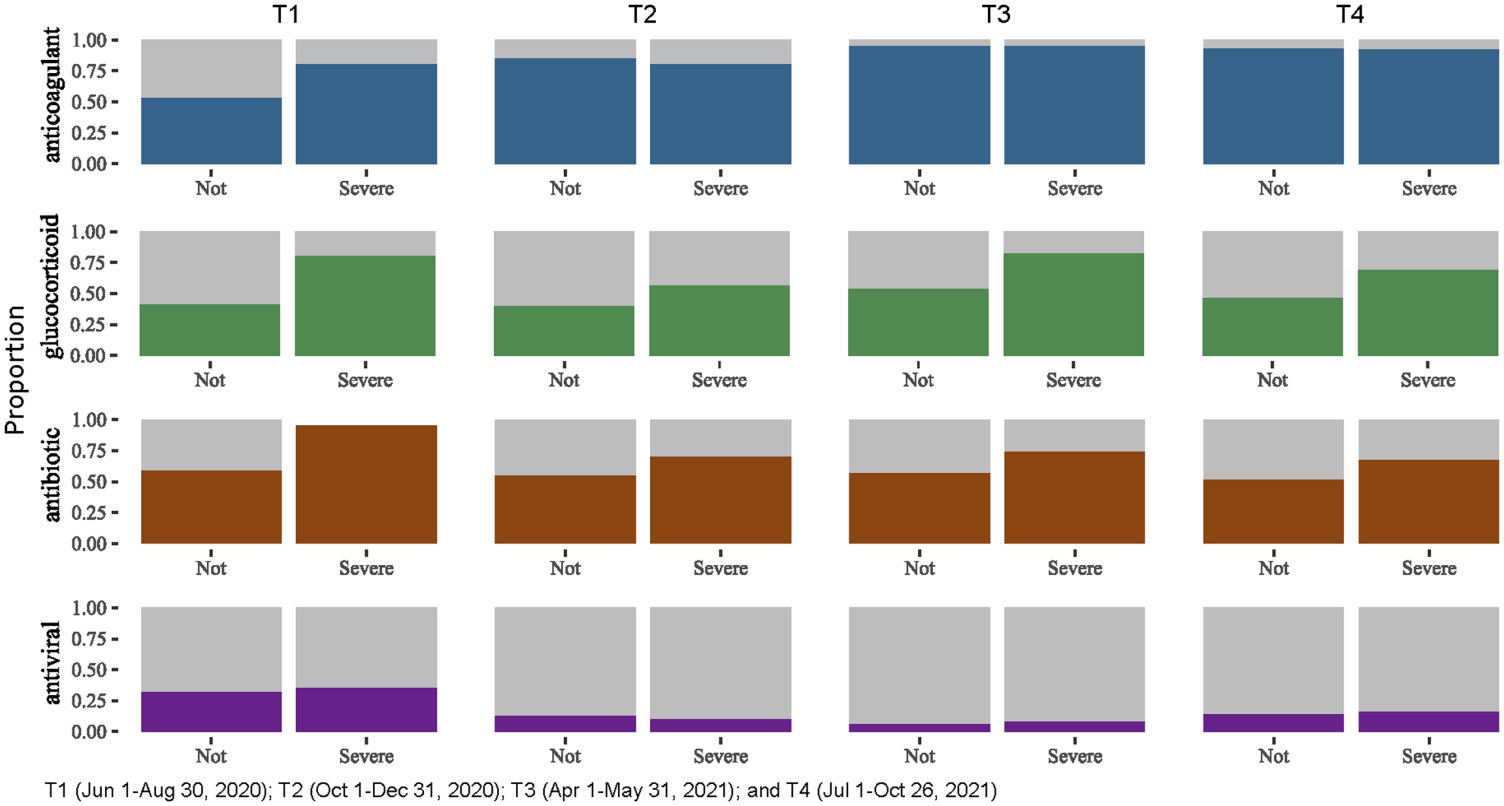
Treatments administered for people hospitalized with severe and non-severe COVID-19, Almaty, Kazakhstan, 2020–2021.

When stratifying treatment types by severe COVID-19 status (Figure 2), we found that proportion of patients receiving glucocorticoid and antibiotics was consistently higher among those with severe COVID-19 across the four time periods.

## Discussion

This study aimed to evaluate the clinical management practices of people hospitalized with COVID-19 in Almaty, Kazakhstan, with respect to the changing national clinical guidance. The results revealed multiple modifications to the national clinical management protocol during the study period involving administration of antibiotic, glucocorticoid, anticoagulant, and antiviral treatments.

Updates to national guidance were published online and distributed to hospitals, some webinars were held on the updates. Although there was some evidence of change in treatment in the time immediately after a guidance update, clinical practice alignment with national guidelines was low across all treatment types. This finding is in contrast with other studies that have found that clinicians followed guidelines and treatment of hospitalized patients was generally responsive to changes in medical evidence and public policy over the course of the early phases of the pandemic (8, 11, 18).

Anticoagulants were the most frequently used treatment in our study. High use of anticoagulants observed in this study reflected the growing recognition of thrombotic complications associated with COVID-19 (19). Notably, half of participants were receiving anticoagulants in T1, even before they were added to national guidelines as a recommended practice for people who were hospitalized with severe COVID-19 in guidelines. Although WHO has never included anticoagulant use in COVID-19 treatment guidelines, several clinical trials had evidence for its use during the early stages of the pandemic. Once added to guidelines proportion of patients receiving anticoagulant treatments increased, but many of these patients did not have a diagnosis of severe COVID-19. nor did they have pulmonary embolism (PE) or deep vein thrombosis (DVT). The high rates of anticoagulant use outside of recommendations are concerning, as thromboprophylaxis are not without risk, and can result in life-threatening bleeding for patients who do not need it (20).

Over half of hospitalized patients in our study were given antibiotics with cephalosporin antibiotics being the most frequently prescribed. These rates are high but below the global estimate of 75% of COVID-19 patients having received antibiotic prescriptions (21). We found that of the antibiotics administered, 95% were used without evidence of secondary infection. High rates of antibiotic misuse raise concerns about the appropriateness of antibiotic use in COVID-19 management. This finding is consistent with other studies highlighting challenges in antimicrobial stewardship during the pandemic (22). Overuse of antibiotics can contribute to antimicrobial resistance, a global public health concern (23, 24). Efforts should be made to ensure judicious use of antibiotics and adherence to evidence-based guidelines.

Glucocorticoids were first added to the national guidelines in end of June 2020. Our study showed an increase in use of glucocorticoids and improvement in alignment with guideline updates over period. This finding is noteworthy, as it indicates a potential learning curve and increasing confidence among clinicians in the use of glucocorticoids as a treatment option for COVID-19. This was ahead of WHO guidance which first added it in September 2020; however, clinical trials had demonstrated utility of corticosteroids before WHO guidance (25). Findings from studies at the time demonstrated the efficacy of dexamethasone in reducing mortality among severely ill COVID-19 patients (25, 26). It underscores the importance of updated evidence-based guidelines to guide clinical practice and improve adherence to guidance.

The low frequencies of antiviral treatment observed in this study align with findings from the Solidarity Trial, which failed to demonstrate significant benefits of specific antiviral therapies in COVID-19 treatment. Studies showed limited effectiveness of drugs like remdesivir and lopinavir/ritonavir in reducing mortality or improving clinical outcomes. Moreover, during T1 to T3 of the study there were no COVID-19 specific antivirals available in country, and in T4 Remdesivir was approved experimentally for COVID-19 with very limited availability in country. The findings from this study reflect clinician cautious use of antivirals in COVID-19 treatment and the limited availability of the drug for treatment.

Interpretation of results are subject to some important limitations. Firstly, the study was conducted in a specific hospital system in Almaty, Kazakhstan, which may limit the generalizability of the findings to other regions or healthcare settings. It is also not reflective of all hospitals in Almaty, where 7 multifunctional city hospitals provided treatment for patients with suspected COVID-19, but these were not included in the study. Secondly the study relies on secondary data routinely input by providers in patient electronic medical records. Data entry errors could have occurred especially in moments of high patient load in healthcare facilities. Omission of information would be the most common error in these scenarios. This would have resulted in an underestimate of treatments. It could also result in overestimation of non-adoption of treatment if patients were not classified correctly as having severe COVID-19. Also, the four time periods of our study do not directly align with dates when changes to guidelines were made, and this can attenuate differences between one period and the next. Lastly, we did not interview providers to assess their level of knowledge of guidelines and their own perceived uptake of COVID-19 treatments.

In conclusion, this study highlights the need for improved adoption of evolving clinical practice guidance for people hospitalized with COVID-19 in Almaty, Kazakhstan. Efforts are needed to enhance communication, education, and support for clinicians to ensure real-time and consistent use of evidence-based treatments, promoting appropriate use of medications, and optimizing patient outcomes as part of any pandemic response.

## Data availability statement

The data analyzed in this study is subject to the following licenses/restrictions: data are from electronic medical records and restricted due to patient confidentiality regulations. A limited deidentified dataset can be obtained from corresponding author. Requests to access these datasets should be directed to SG, sayagazezova@gmail.com.

## Ethics statement

Ethical approval of the study was received from the local Ethical Commission of the NAO Kazakh National Medical University named after N.N. S.D. Asfendiyarov, Kazakhstan [No. 6 (129), 05/25/2022]. This activity was reviewed by the CDC and was conducted consistently with applicable U.S. federal law and CDC policy. See 45 C.F.R. part 46, 21 C.F.R. part 56; 42 U.S.C. §241(d); 5 U.S.C. §552a; 44 U.S.C. §3,501 et seq.

## Author contributions

DN, RH, and MS contributed to conception of the study. DN, RH, and SG organized the data collection and analysis. SG, DN, SZ, and LK contributed design, methods of the study, and interpretation of the data. RH and MS contributed to interpretation of the study findings. SG wrote the first draft of the manuscript. RH, DN, LK, and SZ wrote sections of the manuscript. RH, AD, and DN reviewed the manuscript at all stages. All authors contributed to the article and approved the submitted version.

## Funding

Support for this project was provided by the United States Centers for Disease Control and Prevention, Central Asia Field Epidemiology Training Program (CDC Cooperative Agreement GH15-20-2108).

## Conflict of interest

The authors declare that the research was conducted in the absence of any commercial or financial relationships that could be construed as a potential conflict of interest.

## Publisher’s note

All claims expressed in this article are solely those of the authors and do not necessarily represent those of their affiliated organizations, or those of the publisher, the editors and the reviewers. Any product that may be evaluated in this article, or claim that may be made by its manufacturer, is not guaranteed or endorsed by the publisher.

## Author disclaimer

The findings and conclusions in this report are those of the author(s) and do not necessarily represent the official position of the U. S. Centers for Disease Control and Prevention.
