# Supplementary material for: Therapies for people hospitalized with COVID-19 and alignment with national clinical guidelines in a large hospital, Almaty, Kazakhstan, 2020–2021
Source: Front Med (Lausanne). 2023 Sep 21;10:1248959. doi: 10.3389/fmed.2023.1248959 (PMC10566366; doi:10.3389/fmed.2023.1248959)
Supplement: Supplementary file 1 [file Table_1.pdf]

## Supplementary Table 1. National COVID-19 clinical guidelines updates, Kazakhstan, 2020-21

<table>
<tr><th rowspan="2">Time period</th><th colspan="2">T1</th><th>T2</th><th>T3</th><th>T4</th></tr>
<tr><th colspan="2">Jun 1-Aug 30, 2020</th><th>Oct 1-Dec 31, 2020</th><th>Apr 1-May 31, 2021</th><th>Jul 1-Oct 26, 2021</th></tr>
<tr><td>Guidelines name</td><td>Coronavirus Infection COVID-19 Clinical Protocol, 9th revision</td><td>Coronavirus Infection COVID-19 Clinical Protocol, 10th revision</td><td>Coronavirus infection COVID-19 in adults</td><td>Coronavirus infection (COVID-19) in adults</td><td>Coronavirus infection (COVID-19) in adults</td></tr>
<tr><td>Date</td><td>June 15, 2020</td><td>July 15, 2020</td><td>December 3, 2020</td><td>April 1, 2020</td><td>July 15, 2021</td></tr>
<tr><td>Antibiotics</td><td colspan="5">Broad use not recommended, only for patients' w/ evidence of bacterial infection with severe and extreme severity, the drug of choice is azithromycin</td></tr>
<tr><td>Anticoagulants</td><td>Not in guidelines</td><td>Not in guidelines</td><td colspan="2">Prophylactically for hospitalized patients w/ severe COVID-19; therapeutically for patients with deep vein thrombosis or pulmonary embolism.<br>Dosing:<br>• Nadroparin calcium: Prophylactic dose 0.3-0.4 ml 1x daily; Therapeutic dose 0.4 ml 2x daily<br>• Enoxaparin: Prophylactic dose 0.4 ml 1x daily; Therapeutic dose 0.4 ml 2x daily<br>• Fondaparinux (w/ platelet <100k x109 / l): Prophylactic dose SC 2.5 mg 1x daily<br>• Heparin-Subcutaneously 5000: IU 3 times per day (w/ eGFR <30 ml min)</td><td>Low molecular weight heparins or heparin in therapeutic doses only for hospitalized patients with signs of thrombosis. In the case of diagnosis of deep vein thrombosis or pulmonary embolism should be transferred to a therapeutic dose of unfractionated heparin, low molecular weight heparins, fondaparinux</td></tr>
<tr><td>Glucocorticoids</td><td>Not in guidelines</td><td>Hospitalized patients w/ severe pneumonia</td><td colspan="3">Hospitalized patients w/ severe COVID-19</td></tr>
<tr><td></td><td></td><td colspan="4">Dosing:<br>• Dexamethasone 6 mg orally/intravenously daily, 7-10 days<br>• Methylprednisolone 32 mg orally/intravenously, in 2-3 divided doses (e.g., 8 mg every 6 hours or 16 mg every 12 hours), 7-10 days<br>• Prednisolone 40 mg daily orally, in 1-2 divided doses, 7-10 days</td></tr>
<tr><td>Antivirals</td><td>Not in guidelines</td><td>Not in guidelines</td><td>Not in guidelines</td><td colspan="2">Hospitalized patients with risk factors for severe COVID-19 receiving oxygen and not receiving mechanical ventilation.<br>Dosing as early as possible from symptom onset:<br>• Remdesivir 200 mg IV on day 1 then 100 mg IV daily for 5-10 days</td></tr>
</table>

Source: Ministry of Healthcare of Kazakhstan. Clinical guidelines for diagnosis and treatment of Coronovirus disease, COVID-19 (9th, 10th editions). 2020. Ministry of Healthcare of Kazakhstan. Coronavirus infection COVID-19 in adults (editions by date), 2021.
